# Microwave Effect for Glycosylation Promoted by Solid Super Acid in Supercritical Carbon Dioxide

**DOI:** 10.3390/ijms10125285

**Published:** 2009-12-08

**Authors:** Hiroshi Hinou, Naohiro Saito, Masato Ogawa, Takahiko Maeda, Shin-Ichiro Nishimura

**Affiliations:** 1 Division of Advanced Chemical Biology, Graduate School of Life Science, Hokkaido University, N21, W11, Sapporo 001-0021, Japan; E-Mails: nao-saito@glyco.sci.hokudai.ac.jp (N.S.); drumist@glyco.sci.hokudai.ac.jp (T.M.); 2 Department of Technical Engineering, Faculty of Science, Hokkaido University, N10, W8, Sapporo 060-0021, Japan; E-Mail: m-ogawa@sci.hokudai.ac.jp (M.O.)

**Keywords:** glycosylation, microwave, super solid acid, supercritical carbon dioxide, dielectric constant, electric delocalization, symmetric structure

## Abstract

The effects of microwave irradiation (2.45 GHz, 200 W) on glycosylation promoted by a solid super acid in supercritical carbon dioxide was investigated with particular attention paid to the structure of the acceptor substrate. Because of the symmetrical structure and high diffusive property of supercritical carbon dioxide, microwave irradiation did not alter the temperature of the reaction solution, but enhanced reaction yield when aliphatic acceptors are employed. Interestingly, the use of a phenolic acceptor under the same reaction conditions did not show these promoting effects due to microwave irradiation. In the case of aliphatic diol acceptors, the yield seemed to be dependent on the symmetrical properties of the acceptors. The results suggest that microwave irradiation do not affect the reactivity of the donor nor promoter independently. We conclude that the effect of acceptor structure on glycosylation yield is due to electric delocalization of hydroxyl group and dielectrically symmetric structure of whole molecule.

## 1. Introduction

Since Fischer glycosylation was first used in 1893 [1], acid promoted chemical glycosylation is a central process in the synthesis of glycoside [2]. Despite social demand for a reduction in consumption in chemical processes, the combination of organic solvents and soluble promoters, which are both harmful for the environmental and difficult to separate from other chemicals, were most commonly used in mainstream chemical glycosylation processes [2]. Recently, some pioneering efforts resulted in the replacement of the promoter and solvent of glycosylation process with environmental friendly chemicals, using solid super acids [3,4] and ionic liquids [5]. Because of the increasing importance of glycoconjugates in life science, an environmentally benign and efficient glycosylation method is in high demand for practical and industrial scale production of natural and unnatural glycosides. Natural product origin or enzymatic synthesized glycosides have been recognized as a much more sustainable protocol [6–8]. However, chemical syntheses of the glycoside bond still have a large advantage over other methods due to their structural diversity and functionalization of product oligosaccharides. Chemo–enzymatic synthetic methods (*i.e.*, enzymatic modification of chemically synthesized core oligosaccharide) seem to meet the needs for practical preparation of a large variety of glycoconjugates to be used as bio–probe and drug seeds [9–12]. This means that making the chemical process sustainable is an important requirement. Previously, we have reported a glycosylation process which uses the combination of a solid super acid and supercritical (sc)CO_2_. Not only was this simple and organic solvent–free separation process but it also demonstrated higher reactivity than conventional organic solvents as a result of the good compatibility between surface of the solid acid and the diffusion properties of scCO_2_ [13]. As CO_2_ has a critical point close to ambient temperature (*Tc* = 31 °C, *Pc* = 7.4 MPa) and is nonflammable, nontoxic, inexpensive and scCO_2_ are attractive alternative of common organic solvent.

Microwave (MW) irradiation is also an attractive choice to promote reactions and is an energy effective heating method compared to conventional heat conduction type methods (such as an oil bath) due to the direct heating of the reaction mixture. Additionally, MW irradiation often shows an acceleration of the reaction rate and product selectivity, thereby improving the reaction time and total yield respectively [14]. MW-assisted glycosylations promoted by acid catalysts were also reported for Fischer glycosylation [15], and an acetal exchange reaction from a methyl glycoside type donor [16], glycosyl acetate type donor [17,18], and a trichloroimidate type donor [19]. However MW-assisted glycosylation in a nonpolar solvent such as scCO_2_ has not been reported. Polar solvent are chosen for MW-assisted synthesis because the mechanism of microwave heating is dielectric losses of the irradiated material, and the dielectric constant (ɛ) of the solvent is affected in MW-assisted solution phase synthesis [20]. Both liquid and supercritical CO_2_ have extremely low dielectric constants (ɛ = 1~1.5) [21,22] and scCO_2_ itself is supposed to be inert to MW irradiation. Normally, the MW inert solvent was avoided for MW assisted synthesis without passive heating by the presence a strong MW absorbing material [23]. To the best of our knowledge, MW heating in scCO_2_ was reported only for extraction process to heat solid material directly [24]. However, our previous study suggested a nonthermal effect of MW irradiation for glycosylation processes [17] using temperature controlled MW irradiation by simultaneous cooling [25]. It is interesting how the MW inert and high thermal diffusion properties of scCO_2_ are affected to the MW-assisted reactions. It is believed that the reaction system also show alternative reactivity without thermal elevation.

In this study, we have focused on the MW effects on glycosylation promoted by solid acid in scCO_2_.

## 2. Results and Discussion

**Figure 1.**
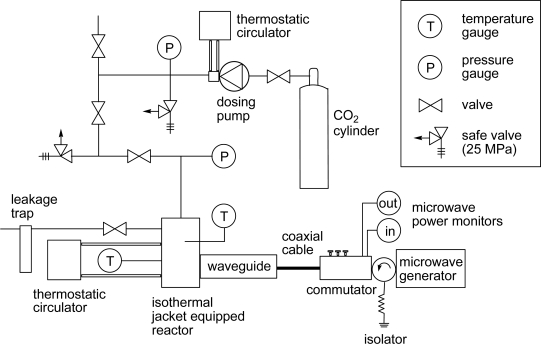
Schematic view of microwave-assisted scCO_2_ reactor.

A novel reaction apparatus equipped with MW controller, liquid CO_2_ controller, thermo controller, and pressure controller was developed and examined in this study. Figure 1 shows schematic view of the apparatus. The MW generator yields 2.45 GHz and ~ 3.0 kW, and single mode MW irradiation was selected for stable radiation into the reactor [25]. The MW absorption efficiency of the reaction solutions was monitored by a MW power monitor. The reaction temperature was regulated by an isothermal jacket on the reactor and was monitored at the alloy wall of the reactor and at the scCO_2_ solution area directly using metal sheathed thermocouples.

As shown in Schemes 2,3,4,6-tetra-*O*-acetyl-α-d-galactopyranose trichloroacetimidate (**1**) [26] was used as a common glycosyl donor substrate in this study. One hundred mg of the glycosyl donor **1** with various glycosyl acceptors (3 equivalents for **1**) were reacted with sulfated zirconia (ZrO_2_/SO_4_; 100 wt% for **1**) under MW irradiation (0 or 200 W) in scCO_2_ (37 °C, 8.0 MPa) for 3 h to give glycoside **3**. All reactions were conducted twice and each yield was calculated from the weight of the product **3** after purification by column chromatography. In all reactions in this study, the temperature of the reaction solutions did not show detectable changes when subject to 200 W of MW irradiation. It is believed that that the dielectric and diffusion properties of scCO_2_ minimize the temperature changes in the solution due to exposure to MW irradiation.

Table 1 indicates the result of the glycosylation reactions with aliphatic alcohols **2a-d**. Without MW irradiation, β-glycoside **3a** [27], **3b**, **3c** [28] and **3d** [28] were isolated in 53~65% yields. With MW irradiation, the yields of **3a-d** showed considerable increase in the 12~26% range. The increasing range of each product due to MW irradiation (**3d**, **3a**, **3b, 3c**) seems to be reflected in the order of molecular polarity of each acceptor.

Table 2 indicates the results of the glycosylation reactions with phenolic alcohols **2e**–**i**. Without MW irradiation, β-glycoside **3e** [29], **3f** [29], **3g** [29], **3h** [30] and **3i** [30] were isolated in 5~43% yields. Among the *p*-substituted acceptors **2e**–**h**, the electrostatic properties of the *p*-substitution groups clearly affected the yields of the glycoside **3e**–**h** that were increased by electron donating substitution groups (-OMe, -Me) and decreased by withdrawing substitution group (-NO_2_) compared to unsubstituted phenyl glycoside **3e**. The order of the yields of the glycoside **3e**–**h** was inverted to the yields of S_N_2 type reactions between α-galactosyl bromide and phenoxides [29]. The position of the substitution group also affected the yield and the *o-*OMe phenol **3i** showed lower yield than *p*-OMe phenol **3h**. In contrast to the aliphatic glycoside **3a**–**d**, obvious effects of MW irradiation were not observed in the yields of all phenolic glycosides **3e**–**i**, which were not affected by the difference in dielectric constant nor the electron density on the phenolic hydroxyl group in **2e**–**i**. Electron delocalization, which is the obvious difference between aliphatic acceptors **2a**–**d** and phenolic acceptors **2e**–**i**, is believed to be the reason for differences between them.

Table 3 indicates the result of the glycosylation reactions with diols **2j**–**n**. Without MW irradiation, monoglycosylated products **3j** [31], **3k** [32], **3l** [32], **3m**, and **3n** were isolated in 6~20% yields. Diglycosylated products were also isolated from the reaction with aliphatic diol acceptors to give **4m** [33] and **4n** in 12 and 17% yields respectively but were not detected from the reaction with phenolic diols **2j**–**l**. In contrast to the methoxyphenyl glycoside **3h** and **3i**, the yield of hydroxyphenyl glycoside **3j** and **3l** showed an increase due to a change in position of the substitution group from *para* to *ortho*. In the case of aliphatic alcohols, the sums of the glycoside yields of **3m** and **4m**, **3n** and **4n** were decreased from the yields of **3d** and **3a** (corresponding products from monools) respectively. Although it made us suspect the solubility of the diols (especially **2m**), it was confirmed that **2m** was dissolved in scCO_2_ under the condition of glycosylation using window equipped reactor [16]. Among the phenolic diols **2j**–**l**, obvious MW effects were not observed in the yields of the monoglycosylated product **3j**–**l** and diglycosylated products also were not obtained. 1,4–Butandiol **2m** showed clear MW effects regarding an increase in the yields of both mono- and diglycosylated product **3m** and **4m** in the 18 and 6% ranges respectively. In the case of *trans*-1,4-cyclohexanediol **2n** which is a conformationally-rigid symmetric diol, however, the MW effect was not observed in the yields of both products **3n** nor **4n**. This unchanged reactivity for the aliphatic diol **2n** indicates that the dielectric inertness by symmetrical conformation also affected to the MW effect. These results also indicate that MW irradiation did not affect the activity of the glycosyl donor **1** or the promoter ZrO_2_/SO_4_, or at least harmonized the activation of acceptors **2** and the donor **1** or promoter were required for increasing reactivity by MW. A polar solvent may also work as a medium for the harmonization of inter-reactants to accelerate reactions under MW irradiation.

## 3. Experimental Section

### 3.1. Materials

Sulfated zirconia (zirconium composition is 60–70%) was purchased from Wako Chemical. The sulfated zirconia was calcined at 600 °C for 2 h before use, and cooled in a glass desiccator without the use of drying agent. All acceptors were purchased in the anhydrous form and added to the reaction mixture without any pretreatment. Advantech No. 2 filter paper was used for the separation of catalysts to stop the reactions. The yield and α/β ratio of the product was calculated from isolated yield and integral area of product anomeric protons using ^1^H-NMR spectroscopy (Bruker AM-500 spectrometer operating at 500.13 MHz), respectively.

### 3.2. General Method for Glycosylation Reactions

2,3,4,6-Tetra-*O*-acetyl-α-d-galactopyranosyl trichroloacetoimidate (**1**, 100 mg, 203 μmol), acceptor **2** (3.0 eq), and ZrO_2_/SO_4_ (100 mg) were added to the reactor, which was heated to 37 °C and CO_2_ was introduced successively from a reservoir by a high–pressure pump. The reaction time was counted from the point at which the CO_2_ pressure reached 8.0 MPa. After the reaction has been sustained at 37 °C and 8.0 MPa for 3 h, CO_2_ was slowly leaked out. The residual solid was washed out with CHCl_3_. ZrO_2_/SO_4_ was removed by filtration. The filtrate was concentrated *in vacuo* and the residue was purified by flash column chromatography to give the glycoside **3**.

*Cyclohexanemethyl 2,3,4,6-tetra-O-acetyl-β-d-galactopyranoside* (**3b**): ^1^H-NMR (500 MHz, CDCl_3_): σ 5.39–5.38 (d, 1H, *J* = 3.3 Hz, H-4), 5.22–5.19 (dd 1H *J* = 8.0, 2.4 Hz, H-2), 5.03–5.00 (dd, 1H, *J* = 7.1, 3.5 Hz, H-3), 4.44–4.42 (d, 1H, *J* = 7.9 Hz, H-1), 4.18–4.12 (m, 2H, H-6), 3.91–3.89 (t, 1H, *J* = 6.8 Hz, H-5), 3.74–3.71 (dd, 1H, *J* = 5.9, 3.5 Hz, CH_2_), 3.26–3.23 (dd, 1H, *J* = 7.1, 2.2, CH_2_), 2.15, 2.05, 2.04, 2.00 (s, 12H, 4 × CH_3_), 1.75–1.66 (m, 6H, CH_2_), 1.62–1.54 (m, 1H, CH), 1.24–1.22 (m, 2H, CH_2_), 0.93–0.90 (m, 2H, CH_2_); 13C-NMR (125 MHz, CDCl_3_): σ 20.60, 25.71, 25.78, 26.51, 29.54, 29.70, 37.80, 61.31 (C-6), 67.27 (C-4), 69.03 (C-2), 70.59 (C-5), 71.06 (C-1), 75.96, 101.7 (C-1), 168.61, 170.00, 170.24, 170.44; HRMS (ESI-TOF) *m/z*: calculated for C_21_H_32_O_10_Na: 467.18877, found: 467.18950 [M + Na]^+^.

*4-Hydroxybutyl 2,3,4,6-tetra-O-acetyl-β-d-galactopyranoside* (**3m**): ^1^H-NMR (500 MHz, CDCl_3_): σ 5.39–5.38 (d, 1H, *J* = 3.3 Hz, H-4), 5.22–5.18 (dd, 1H, *J* = 8.2, 2.4 Hz, H-2), 5.03–5.01 (dd, 1H, *J* = 7.1, 3.4 Hz, H-3), 4.48–4.47 (d, 1H, *J* = 7.9 Hz, H-1), 4.21–4.11 (m, 2H, H-6), 3.96–3.94 (m, 1H, CH), 3.92–3.90 (t, 1H, *J* = 6.7 Hz, H-5), 3.66–3.64 (td, *J* = 4.6, 1.5 Hz, 2H, CH_2_), 3.55–3.51 (m, 1H, CH), 2.16, 2.06, 2.05, 1.99 (s, 12H, 4 × CH_3_), 1.73–1.58 (m, 4H, 2 × CH_2_); 13C-NMR (125 MHz, CDCl_3_): σ 20.59, 20.67, 20.77, 25.89, 29.33, 61.32 (C-6), 62.37, 67.11 (C-4), 68.95 (C-2), 70.00, 70.69 (C-5), 70.95 (C-3), 101.32 (C-1), 169.55, 170.20, 170.31, 170.44; HRMS (ESI-TOF) *m/z*: calculated for C_18_H_28_O_11_Na:443.15293, found:443.15218 [M + Na]+.

*Trans-4-Hydroxycyclohexyl 2,3,4,6-tetra-O-acetyl-β-d-galactopyranoside* (**3n**): ^1^H-NMR (600 MHz, CDCl_3_): σ 5.37 (s, 1H, Hz, H-4), 5.21–5.18 (t, 1H, *J* = 9.1 Hz, H-2), 5.03–5.00 (dd, 1H, *J* = 10.6, 3.3 Hz, H-3), 4.56–4.52 (d, 1H, *J* = 8.8 Hz, H-1), 4.24–4.04 (m, 2H, H-6), 3.89–3.87 (t, 1H, *J* = 6.4 Hz, H-5), 3.81–3.76 (m, 1H, CH), 3.72–3.66 (m, 1H, CH), 2.15, 2.05, 2.03, 1.99 (s, 12H, 4 × CH_3_), 1.88–1.45 (m, 9H, OH + 4 × CH_2_); 13C-NMR (150 MHz, CDCl_3_): σ 22.10, 22.17, 22.27, 22.33, 28.56, 30.57, 31.52, 31.93, 62.80 (C-6), 68.62 (C-4), 69.76, 70.56 (C-2), 72.10 (C-5), 72.53 (C-3), 75.65, 101.03 (C-1), 170.84, 171.73, 171.86, 171.94; HRMS (ESI-TOF) *m/z*: calculated for C_20_H_30_O_11_Na: 469.16803, found: 469.16935 [M + Na]^+^.

*Trans-1,4-Bis((2,3,4,6-tetra-O-acetyl-β-d-galactopyranosyl)oxy)cyclohexane* (**4n**): ^1^H-NMR (500 MHz, CDCl_3_): σ 5.39–5.38 (d, 2H, *J* = 3.1 Hz, 2 × H-4), 5.21–5.17 (t, 2H, *J* = 7.9, 2.5 Hz, 2 × H-2), 5.03–5.01 (dd, 2H, *J* = 7.0, 3.4 Hz, 2 × H-3), 4.55–4.53 (d, 2H, *J* = 7.9 Hz, 2×H-1), 4.20–4.06 (m, 4H, 2 × H-6), 3.91–3.86 (t, 2H, *J* = 6.2 Hz, 2 × H-5), 3.81–3.53 (m, 2H, 2 × CH), 2.20–1.95 (s, 24H, 8 × CH_3_), 1.94–1.36 (m, 8H, 4 × CH_2_); 13C-NMR (125 MHz, CDCl_3_): σ 20.61, 20.69, 20.70, 20.75, 26.32, 28.31, 61.24 (C-6), 67.08 (C-4), 68.85 (C-2), 70.60 (C-5), 70.96 (C-3), 73.78, 98.73 (C-1), 169.49, 170.20, 170.32, 170.41; HRMS (ESI-TOF) *m/z*: calculated for C_34_H_48_O_20_Na:799.26311, found:799.26354 [M + Na]^+^.

## 4. Conclusions

The effect of MW on glycosylation promoted by ZrO_2_/SO_4_ in scCO_2_ was investigated with a special focus on the differences between acceptor substrates. The temperature of the reaction mixture was not affected by MW irradiation because of the extremely low dielectric constant and diffusion properties of scCO_2_, which made possible the monitoring of the MW effect under minimized conditions of thermal and solvation effects. The changing range of glycosylation yield between a MW irradiated reaction and a non-microwave reaction clearly showed phenolic and aliphatic acceptors as the unaffected and affected groups, respectively. The electron delocalization of phenolic hydroxyl groups was associated with the cause of the unresponsive properties by MW. The difference of MW effects among aliphatic hydroxyl groups indicated the dielectric neutralization by symmetric conformation also related to it. A harmonized activation mechanism of acceptors **2** and the donor **1** or promoter by MW was also suggested as a cause of the non-thermal MW effect. Additional study of MW effect focused on the dielectric property of the glycosyl donor and promoter and the presence of their harmonizing effect with acceptors are under investigation.

## Figures and Tables

**Scheme 1.**
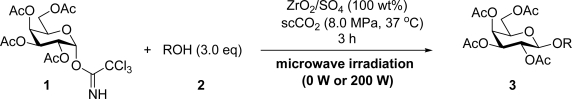
Glycosylation conditions in this study.

**Table 1.** Glycosylation of **1** and aliphatic glycosyl acceptors **2a**–**d** promoted by ZrO_2_/SO_4_ in scCO_2_ with or without MW irradiation.

**Entry**	**Acceptor 2**	**MW (W)**	**Glycoside 3**	**Yield [1^st^, 2^nd^] (%)**
1	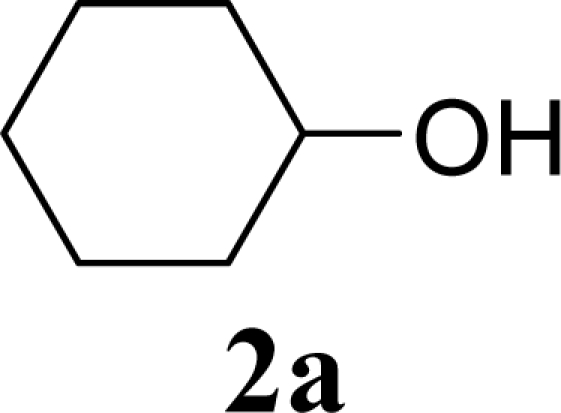	0	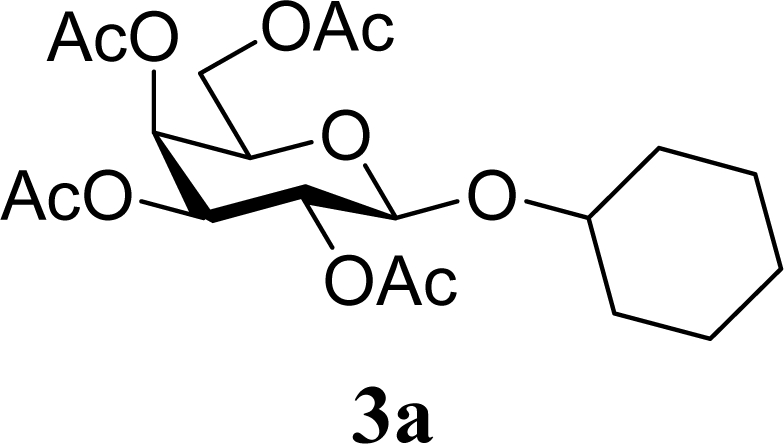	53 [51,55]
2	**2a**	200	**3a**	71 [69,72]
3	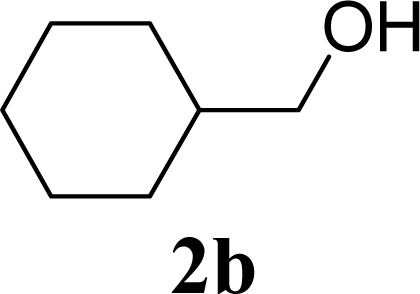	0	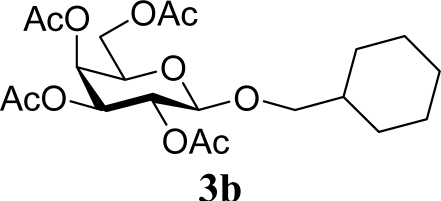	54 [52,55]
4	**2b**	200	**3b**	68 [68,67]
5	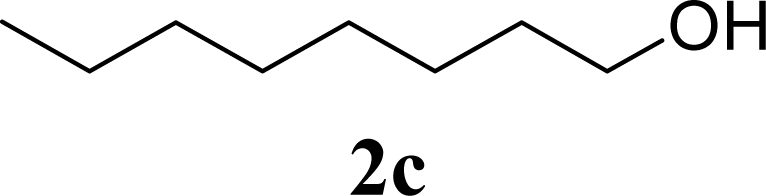	0	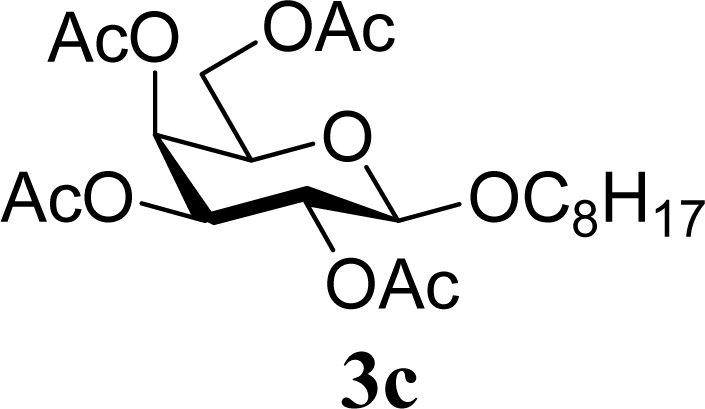	65 [68,62]
6	**2c**	200	**3c**	77 [80,74]
7	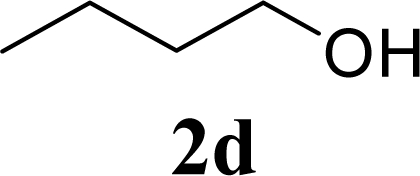	0	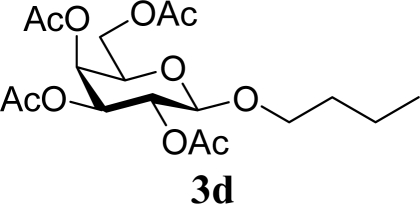	60 [58,61]
8	**2d**	200	**3d**	86 [84,87]

**Table 2.** Glycosylation of **1** and phenolic glycosyl acceptors **2e**–**i** promoted by ZrO_2_/SO_4_ in scCO_2_ with or without MW irradiation.

**Entry**	**Acceptor 2**	**MW (W)**	**Glycoside 3**	**Yield [1^st^, 2^nd^] (%)**
9	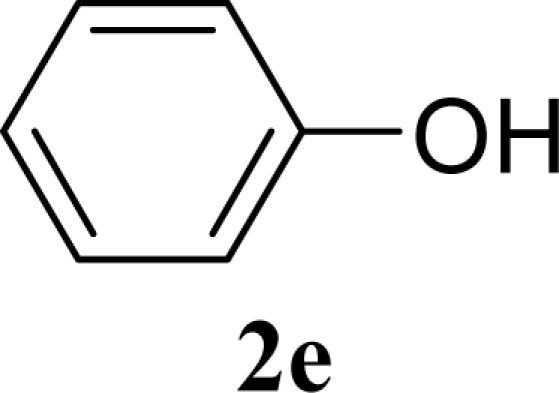	0	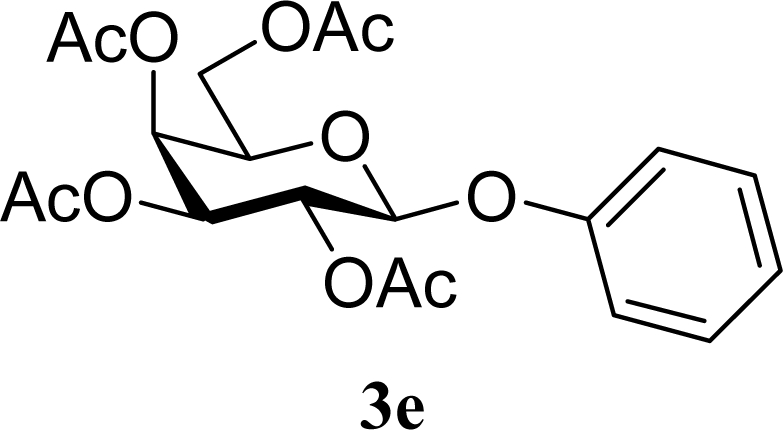	14 [12,15]
10	**2e**	200	**3e**	20 [17,23]
11	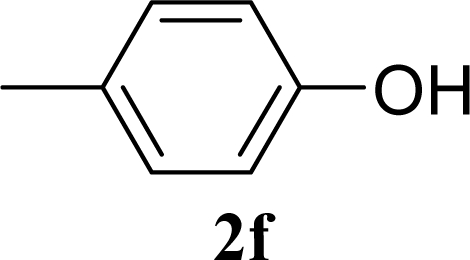	0	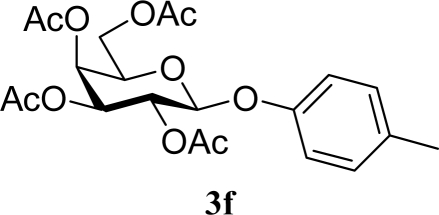	41 [49,33]
12	**2f**	200	**3f**	39 [39,38]
13	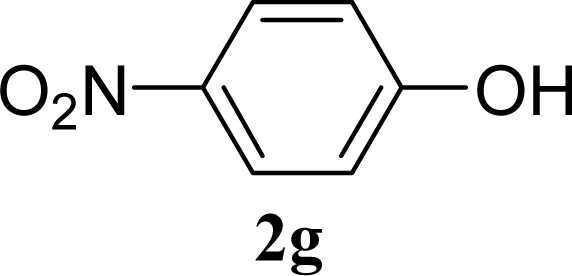	0	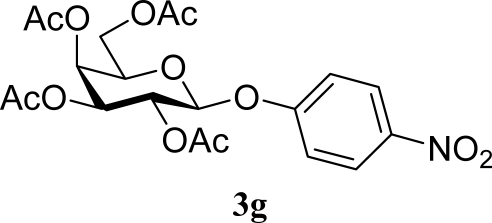	5 [6,4]
14	**2g**	200	**3g**	3 [5,1]
15	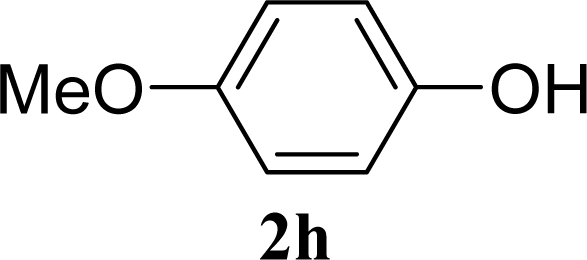	0	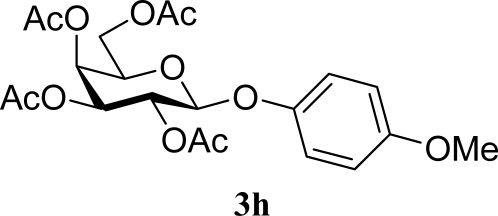	43 [42,44]
16	**2h**	200	**3h**	40 [42,38]
17	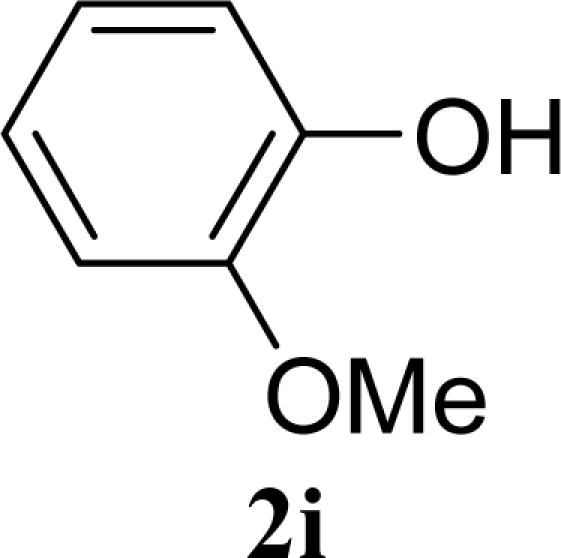	0	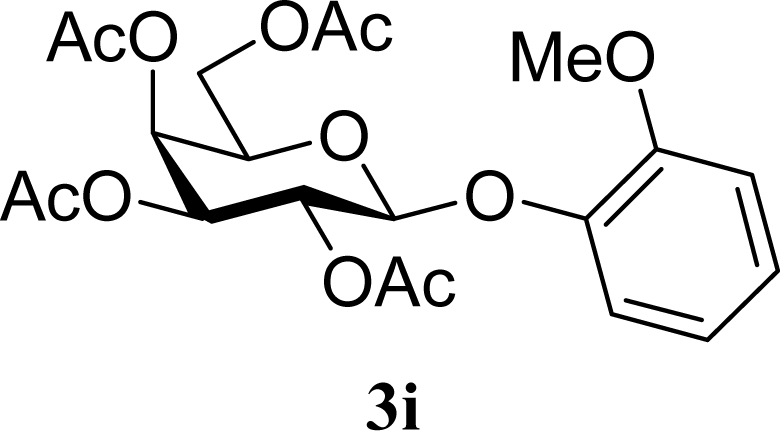	24 [26,22]
18	**2i**	200	**3i**	22 [24,20]

**Table 3.** Glycosylation of **1** and diol-type glycosyl acceptors **2j**–**n** promoted by ZrO_2_/SO_4_ in scCO_2_ with or without MW irradiation.

**Entry**	**Acceptor 2**	**MW (W)**	**Monoglycosylated product 3**	**Yield of 3 [1^st^, 2^nd^] (%)**	**Yield of diglycosylated product 4 (%)**
19	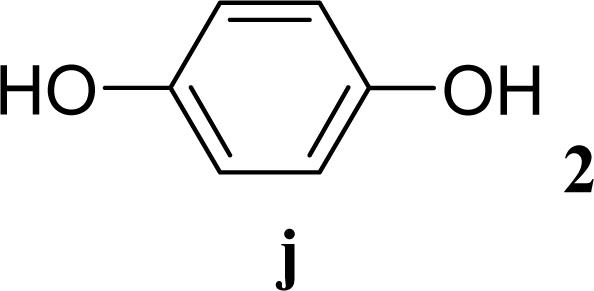	0	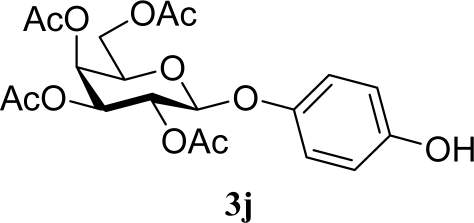	6 [7,4]	n. d.
20	**2j**	200	**3j**	5 [4,6]	n. d.
21	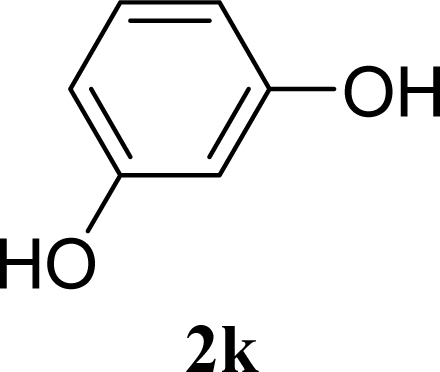	0	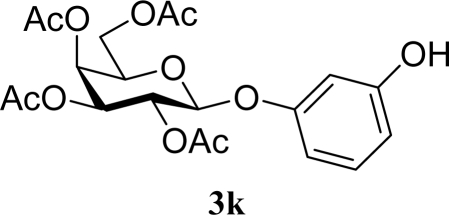	14 [8,19]	n. d.
22	**2k**	200	**3k**	19 [10,28]	n. d.
25	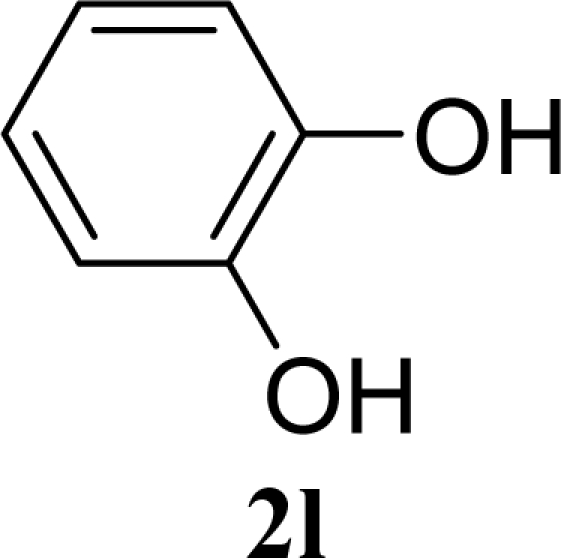	0	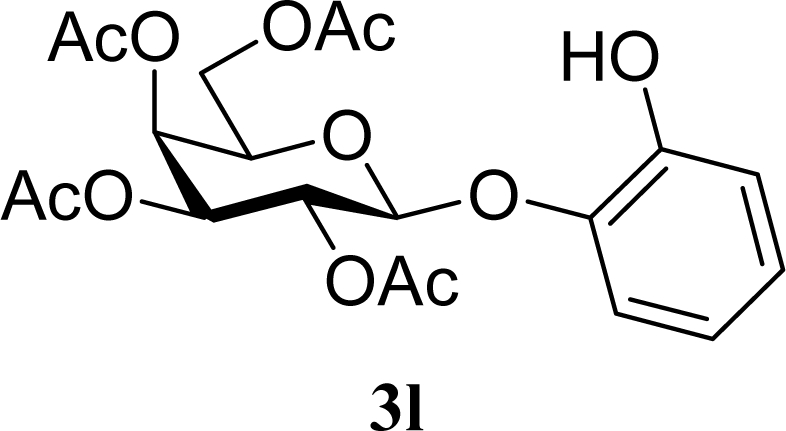	20 [19,21]	n. d.
26	**2l**	200	**3l**	17 [16,18]	n. d.
27	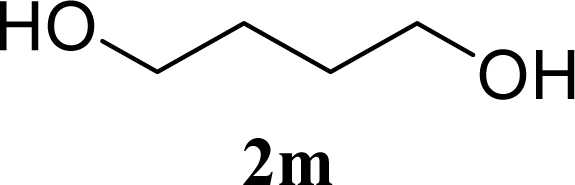	0	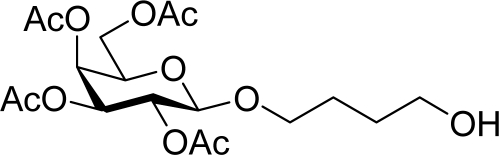	12 [12,12]	**4m**^a^,^b^ 12 [11,12]
28	**2m**	200	**3m**	30 [33,26]	**4m**^a^,^b^
29	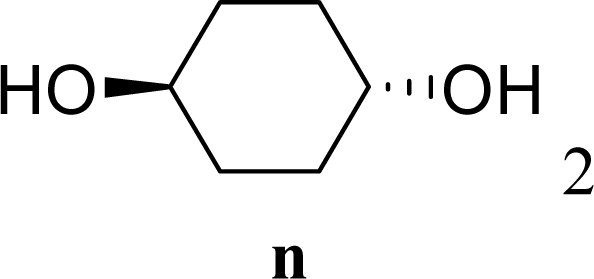	0	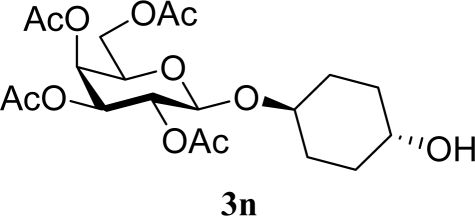	17 [14,20]	18 [16,20] **4n**^a^,^b^
30	**2n**	200	**3n**	21 [23,19]	17 [15,18] **4n**^a^,^b^ 14 [10,17]

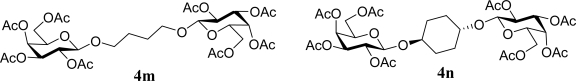

a Structure of diglycosylated product **4m** and **4n**.

b The yields of 4m and 4n were calculated as one half molar equivalent of the glycosyl donor **1** was 100%.
